# Pb-214/Bi-214-TCMC-Trastuzumab inhibited growth of ovarian cancer in preclinical mouse models

**DOI:** 10.3389/fchem.2023.1322773

**Published:** 2024-01-25

**Authors:** Abdullah Metebi, Nathan Kauffman, Lu Xu, Satyendra Kumar Singh, Chelsea Nayback, Jinda Fan, Nathan Johnson, John Diemer, Terry Grimm, Mike Zamiara, Kurt R. Zinn

**Affiliations:** ^1^ Institute for Quantitative Health Science and Engineering, Michigan State University, East Lansing, MI, United States; ^2^ Comparative Medicine and Integrative Biology, Michigan State University, East Lansing, MI, United States; ^3^ Radiological Sciences Department, Taif University, Taif, Saudi Arabia; ^4^ Biomedical Engineering, Michigan State University, East Lansing, MI, United States; ^5^ Department of Chemistry, Michigan State University, East Lansing, MI, United States; ^6^ Radiology, Michigan State University, East Lansing, MI, United States; ^7^ Niowave Inc., Lansing, MI, United States; ^8^ Small Animal Clinical Sciences, Michigan State University, East Lansing, MI, United States

**Keywords:** ovarian cancer, Lead-214/Bismuth-214 (Pb-214/Bi-214), targeted alpha-particle therapy, Trastuzumab, preclinical mouse model

## Abstract

**Introduction:** Better treatments for ovarian cancer are needed to eliminate residual peritoneal disease after initial debulking surgery. The present study evaluated Trastuzumab to deliver Pb-214/Bi-214 for targeted alpha therapy (TAT) for HER2-positive ovarian cancer in mouse models of residual disease. This study is the first report of TAT using a novel Radon-222 generator to produce short-lived Lead-214 (Pb-214, t_1/2_ = 26.8 min) in equilibrium with its daughter Bismuth-214 (Bi-214, t_1/2_ = 19.7 min); referred to as Pb-214/Bi-214. In this study, Pb-214/Bi-214-TCMC-Trastuzumab was tested.

**Methods:** Trastuzumab and control IgG antibody were conjugated with TCMC chelator and radiolabeled with Pb-214/Bi-214 to yield Pb-214/Bi-214-TCMC-Trastuzumab and Pb-214/Bi-214-TCMC-IgG1. The decay of Pb-214/Bi-214 yielded α-particles for TAT. SKOV3 and OVAR3 human ovarian cancer cell lines were tested for HER2 levels. The effects of Pb-214/Bi-214-TCMC-Trastuzumab and appropriate controls were compared using clonogenic assays and in mice bearing peritoneal SKOV3 or OVCAR3 tumors. Mice control groups included untreated, Pb-214/Bi-214-TCMC-IgG1, and Trastuzumab only.

**Results and discussion:** SKOV3 cells had 590,000 ± 5,500 HER2 receptors/cell compared with OVCAR3 cells at 7,900 ± 770. *In vitro* clonogenic assays with SKOV3 cells showed significantly reduced colony formation after Pb-214/Bi-214-TCMC-Trastuzumab treatment compared with controls. Nude mice bearing luciferase-positive SKOV3 or OVCAR3 tumors were treated with Pb-214/Bi-214-TCMC-Trastuzumab or appropriate controls. Two 0.74 MBq doses of Pb-214/Bi-214-TCMC-Trastuzumab significantly suppressed the growth of SKOV3 tumors for 60 days, without toxicity, compared with three control groups (untreated, Pb-214/Bi-214-TCMC-IgG1, or Trastuzumab only). Mice-bearing OVCAR3 tumors had effective therapy without toxicity with two 0.74 MBq doses of Pb-214/Bi-214-TCMC-trastuzumab or Pb-214/Bi-214-TCMC-IgG1. Together, these data indicated that Pb-214/Bi-214 from a Rn-222 generator system was successfully applied for TAT. Pb-214/Bi-214-TCMC-Trastuzumab was effective to treat mouse xenograft models. Advantages of Pb-214/Bi-214 from the novel generator systems include high purity, short half-life for fractioned therapy, and hourly availability from the Rn-222 generator system. This platform technology can be applied for a variety of cancer treatment strategies.

## 1 Introduction

Ovarian cancer is one of the deadliest malignancy of the reproductive system, with 12,810 deaths in the United States in 2022 (Siegel et al., 2022). Over 70% of women with ovarian cancer at diagnosis have cancer spread throughout their peritoneal cavity (Chien and Poole, 2017). Ovarian cancer is known as a “silent killer” as symptoms are missing until late in the disease (Momenimovahed et al., 2019). After diagnosis, the standard of care for ovarian cancer is surgical cytoreduction followed by chemotherapy. The 5-year average survival rate for all types of ovarian cancer is ∼50% and has not improved significantly over time because ovarian cancer becomes resistant to chemotherapy (Bristow et al., 2002; Harter et al., 2008; Elattar et al., 2011; Motohara et al., 2019). There is a pressing need for better treatment options, including strategies to treat residual disease after initial debulking surgery.

Overexpression of human epidermal growth factor receptor 2 (HER2) has been demonstrated in 20%–30% of ovarian cancers, particularly in cancers with poor prognosis (Lanitis et al., 2012; Iqbal and Iqbal, 2014). HER2 plays a critical role in tumor cell survival, growth, differentiation, and metastasis (Moasser, 2007). The FDA-approved humanized monoclonal antibody Trastuzumab (Herceptin) was a significant advancement 25 years ago to block HER2 signaling as a treatment for HER2-expressing metastatic breast cancer. Drug conjugates with Trastuzumab are new approaches for HER2-expressing cancer, for example, Enhertu was FDA approved in 2022 for second-line treatment of breast cancer (Hurvitz et al., 2023); it was also approved for certain gastric and lung cancers (Li et al., 2022; Rha and Chung, 2023).

HER2 expression on ovarian cancer cells offered an opportunity for TAT as a treatment strategy (Nahta and Esteva, 2006). Early demonstrations of TAT to significantly prolong mouse survival using Bi-213- or At-211-immunoconjugates in peritoneal disease models (Bloechl et al., 2005; Elgqvist et al., 2006) were followed by successful TAT using Th-227-DOTA-*p*-benzyl-Trastuzumab in HER2-positive mouse ovarian cancer models (Heyerdahl et al., 2012). More recently, Chung et al. reported a pretargeting TAT strategy based on HER2 targeting in a mouse model and utilizing α-emitting Ac-225. The α particles were efficient in targeting small cancerous regions and even individual cancer cells. This treatment approach significantly extended the median survival of nude mice with SKOV3 peritoneal tumors (Chung et al., 2023).

Pb-212-TCMC-Trastuzumab for TAT was further supported by results from preclinical models of peritoneal cancer (Milenic et al., 2010; Yong et al., 2012; 2013; 2014), acceptable toxicology studies in mice (Milenic et al., 2015), and dosimetry in macaques (Kasten et al., 2016). A phase I clinical trial was conducted in HER2-positive peritoneal disease, primarily ovarian cancer patients (Meredith et al., 2018). This dose-escalation study using IP-dosed Pb-212-TCMC-Trastuzumab was the first-in-human clinical trial to show effective TAT for HER2-positive peritoneal cancer without toxicity (Meredith et al., 2018). TAT with Pb-212 is attractive because of the many excellent generator systems to produce Pb-212, the excellent TCMC chelator to tightly bind Pb-212 to targeting molecules, and the availability of Pb-203 for imaging studies (McNeil et al., 2021; McNeil et al., 2023). The combined approach of Pb-203 imaging followed by Pb-212 therapy fits the radiotheranostic paradigm.

A TAT-based radiopharmaceutical drug has two parts: 1) a cancer targeting component such as a small molecule, peptide, or monoclonal antibody with high affinity for receptors expressed specifically on cancer cells, and 2) an α-emitting radioisotope bound to the targeting component with a chelator molecule (Kauffman et al., 2023a). The α-emitting radioisotopes with potential thus far include Ac-225 (Kratochwil et al., 2016), At-211 (Orozco et al., 2013; Green et al., 2015), Bi-212 (Kauffman et al., 2023b), Bi-213 (Autenrieth et al., 2018), Pb-212 (Meredith et al., 2014; Meredith et al., 2018), Ra-223 (Parker et al., 2018), and Th-227 (Poty et al., 2018b; a). The α-particles are 2+ charged helium nuclei with high kinetic energies (5–9 MeV) and short penetrating ranges in tissue (50–100 μm), resulting in high linear energy transfer (LET) *in vivo*. When specifically targeted to cancer, the short range of α-particles minimizes damage to surrounding healthy, normal cells. In contrast, β-particles are energetic electrons (40–2,300 keV) with lower LET that travel 0.05–12 mm in tissues. Thus, many β-particles are needed to achieve the same dose compared to α-particles, and β-particles have more non-specific targeting to healthy adjacent tissues, which makes radioisotopes that decay exclusively by β-emissions potentially less effective in cancer therapy (Navalkissoor and Ashley Grossman, 2019).

Our current study evaluated Pb-214/Bi-214 from an innovative generator system based on Radon-222 (Rn-222), collected from the natural decay of long-lived Ra-226. The Rn-222 has a 3.8-day half-life and is in the form of a gas. The Pb-214/Bi-214 have relatively short half-lives (Pb-214: t_1/2_ = 26.8 min, Bi-214: t_1/2_ = 19.7 min). The short half-lives make Pb-214/Bi-214 potentially better for fractionated therapy compared to other α-particle-emitting radionuclides. Radiation dose fractionation involves delivering radiation in multiple smaller doses over time. Pb-214/Bi-214 may be advantageous in fractionated therapy by allowing the delivery of therapeutic doses while minimizing damage to normal tissues during each treatment session. Off-target toxicity may also be reduced because the Pb-214/Bi-214 decays before it can accumulate in normal tissues like the kidneys. Prior studies indicated that short-lived Bi-213 (t_1/2_ = 45 min) had reduced toxicity compared with an identical targeting approach using longer-lived Lu-177 (Seidl et al., 2011). In this study, Pb-214/Bi-214 was evaluated as an alternative to Pb-212 (t_1/2_ = 10 h) that had dose-limiting toxicities with 0.3 MBq of Pb-212-labeled antibodies (Kasten et al., 2017; Kasten et al., 2018a; Kasten et al., 2018b). The Trastuzumab was radiolabeled with Pb-214/Bi-214 using the bifunctional chelator TCMC. The applications of Pb-214/Bi-214-TCMC-Trastuzumab for therapeutic studies are reported.

## 2 Materials and methods

### 2.1 Reagents and instruments

Trastuzumab-anns (KANJINTI, biosimilar to Herceptin) was purchased from Amgen (Thousand Oaks, CA). Human IgG1 Kappa-UNLB was obtained from Southern Biotech Inc. (Birmingham, AL). S-2-(4-Isothiocyanatobenzyl)-1,4,7,10-tetraaza-1,4,7,10-tetra(2-carbamoylmethyl)cyclododecane (p-SCN-Bn-TCMC and in manuscript abbreviated as just TCMC) was purchased from Macrocyclics (Plano, TX).

The Pierce™ Protein Concentrator PES, 30 K MWCO, 2–6 mL, and the Zeba desalting column 40 K MWCO, 2 mL were obtained from Thermo Fisher Scientific Inc. (Waltham, MA). Pb-214/Bi-214 radioisotopes from the Rn-222 generator systems were supplied by Niowave Inc. (Lansing, MI). The chromatography strips used were TEC-Control Chromatography Strips, Model# 150-772 (BIODEX, Shirley, NY). The IVIS Spectrum imaging system and Wizard2 Gamma Counter were obtained from Perkin Elmer (Waltham, MD). Radiation safety and IACUC approvals were obtained prior to starting experiments.

### 2.2 Radiolabeling

#### 2.2.1 Conjugation of antibodies with hydrazine nicotinate (HYNIC) and radiolabeling with Tc-99m

A vacuum-dried kit (stored at −80°C) containing carefully measured amounts of HYNIC was resuspended in Na_2_HPO_4_ buffer (0.15 M, pH = 7.4). After dissolving the HYNIC in the vial, it was added to dialyzed Trastuzumab-anns (HYNIC:Trastuzumab; 6:1 M ratio), and the solution was mixed by pipetting. The vial was then covered in aluminum foil to keep in the dark during incubation at room temperature for 1 h with gentle mixing on a rocker. The sample was dialyzed overnight against 1 L PBS (pH = 7.4) buffer to remove any unreacted HYNIC.

For radiolabeling, 1,850 MBq Tc-99m peretechnetate (Cardinal Health, Flint, MI) was added to a kit containing Tricine/SnCl_2_ (36 mg/0.05 mg) and incubated at room temperature for 15 min; then, the Tc-99m/Tricine/SnCl_2_ mixture was added to the Trastuzumab-HYNIC conjugate and incubated for 45 min at room temperature in the dark. Tc-99m radiolabeled Trastuzumab was purified using a G25 resin column, and the purity (Tc-99m bound) was checked by iTLC using 2 different solvents.

#### 2.2.2 Trastuzumab-TCMC and IgG1-TCMC conjugations

Separately, each antibody in PBS buffer was exchanged to carbonate buffer, pH 9. The buffer exchange used a Pierce™ Protein Concentrator. The sample was centrifuged at 3,000–4,000 g for 8 min. Protein concentrations were determined by Bradford protein assay. 1 mg of each antibody was added to a TCMC premade kit (TCMC: Ab molar ratio = 6:1), followed by incubation for 2 h at room temperature. To remove the unconjugated chelator from the TCMC-conjugated antibody, the buffer was exchanged with 0.15 M ammonium acetate buffer, pH 7.0, using the Pierce™ Protein Concentrator PES and centrifuged at 3,000–4,000 g for 8 min. Trastuzumab-anns-TCMC and IgG-TCMC were stored at 4°C.

#### 2.2.3 Rn-222 generator for Pb-214/Bi-214 and radiolabeling Trastuzumab

**FIGURE 1:**
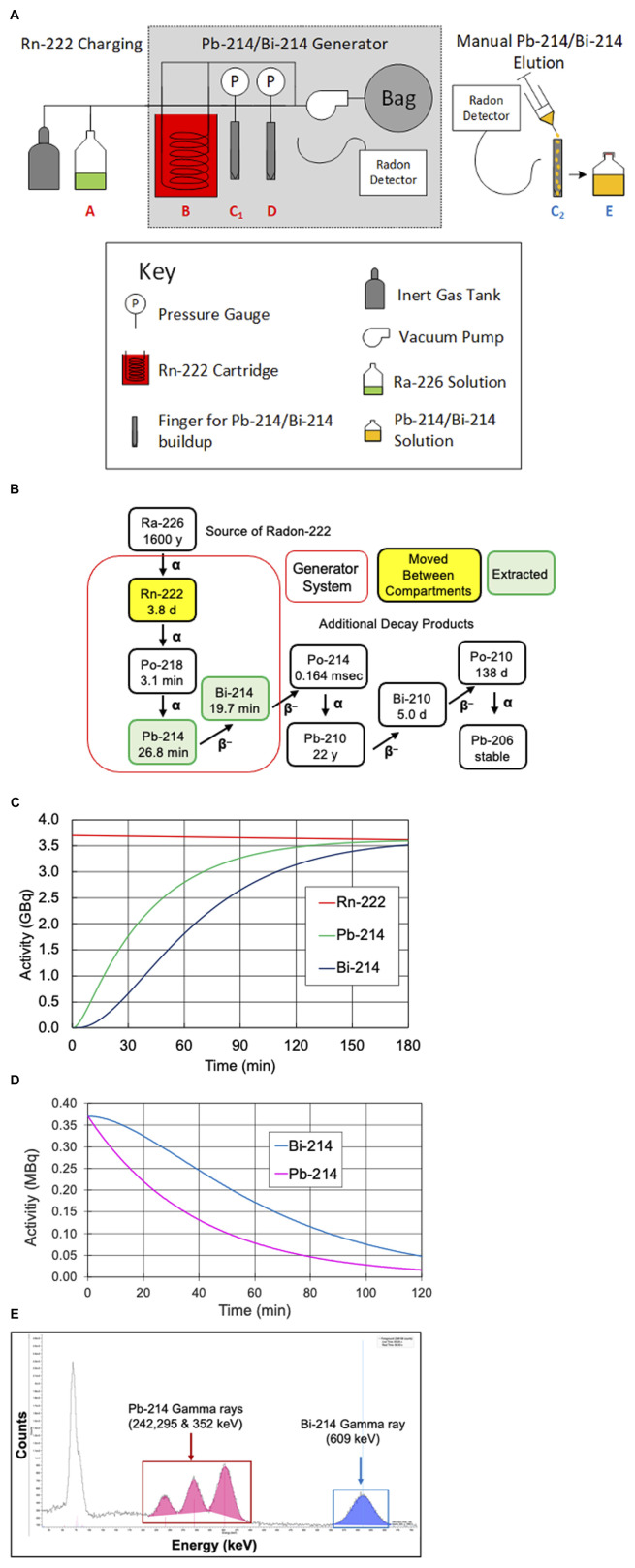
**(A)** Diagram of the manual Rn-222 generator used to produce Pb-214 and Bi-214, **(B)** Rn-222 decay scheme, including the parent radionuclide Ra-226 and showing the generation and extraction of short-lived Pb-214 and Bi-214, **(C)** in-growth of Pb-214 and Bi-214 over time when Rn-222 is moved to C1 or D, **(D)** Pb-214 and Bi-214 decay plot of Pb-214 and daughter Bi-214 over time during equilibrium, and **(E)** gamma-ray spectra of Pb-214 and Bi-214 on a NaI detector.

Figure 1A presents a diagram of the manual generator developed to produce Pb-214/Bi-214. As shown in Figure 1A, Rn-222 was moved between vessels (points A, B, C1, and D) by cryo-cooling the destination and warming the initial location. For our experiments, the Rn-222 was allowed to be present in vessel C1 for 2 h, and was then transferred to vessel D. Subsequently, vessel C1 was removed (with shielding) and Pb-214/Bi-214 was rinsed off the walls (labeled as C2) to produce the Pb-214/Bi-214 solution (point E). The valves are not shown in Figure 1A.

Figure 1B presents the radioisotopes that are part of the generator system, together with their half-lives. Figure 1C shows the in-growth of Pb-214 and Bi-214 over time in a generator system containing 3.7 GBq of Rn-222. With 54 min of Rn-222 decay (two Pb-214 half-lives), 75% of the theoretically maximum of Pb-214 would be available. As shown, increasing the time for Rn-222 decay would result in higher activity levels of the Pb-214 and Bi-214 until equilibrium would be reached, at which time the decay of Pb-214 would equal the in-growth from decay of Rn-222. At equilibrium (∼3 h), the production of Pb-214 (from Rn-222 decay) would be equal to the Pb-214 decay, and the Pb-214/Bi-214 ratio would be equal to one.

Figure 1D shows the decay of equal levels of 0.38 MBq each of Pb-214 and Bi-214 in the absence of Rn-222. These amounts were similar to the dose levels given to the mice. Because Pb-214 decays to Bi-214, the amount of Bi-214 does not appear to follow the 20-min half-life decay. In our studies, the levels of Pb-214 and Bi-214 were determined by their different gamma-ray emissions, for example, by the gamma emissions detected from the NaI detector in the gamma counter, as shown in Figure 1E.

Pb-214 and Bi-214 were collected from the manual generator, as shown in Figure 1A-position C2 with 0.1 M HCl. Representative samples were saved for analysis of chemical impurities by ICP-OES. The radioactivity levels of Pb-214 and Bi-214 and any radioactive impurities were precisely measured using a calibrated Ortec High Purity Germanium (HPGe) radiation detector and a gamma-ray spectroscopy system. The HPGe detector was calibrated using a National Institute of Standards and Technology (NIST) traceable Eu-152 source in a similar geometry. Pb-214 was quantified using the 351.93 keV gamma photon, and Bi-214 was quantified using the 609.31 keV gamma photon (Figures 1B, C). The Pb-214/Bi-214 solution was delivered by courier from Niowave, Inc. (Lansing, MI). The pH was adjusted to 5.5–6.5 (using pH paper, range 2–9) by adding 0.2 mL of 5 M ammonium acetate, pH 7.0, and the sample was vortexed for 3 s. After Pb-214/Bi-214 was neutralized and the pH was adjusted to 5.0–5.5, it was separated into two vials with 70–140 MBq each. One vial was added to the Trastuzumab-anns-TCMC and the second vial to IgG1-TCMC, followed by vortexing for 3 s and incubation for 15 min at 37°C.

#### 2.2.4 Purification of radiolabeled antibodies

Purification was performed using a Zeba desalting column (40 K MWCO, 2 mL). PBS was used as an eluent. The column was prepared by exchanging the buffer using PBS three times. The radiolabeled Trastuzumab-anns or IgG1 was added to the zeba column and centrifuged for 4 min.

#### 2.2.5 iTLC assay

Instant thin layer chromatography (iTLC) was conducted on the purified, radiolabeled antibodies. Samples of the radiolabeled antibody (2 μL each) were placed at the bottom of two strips (TEC-Control Chromatography Strips, Model# 150-772, BIODEX, Shirley, NY), and one strip was placed in a glass vial containing 0.4 mL of 10 mM EDTA in 0.15 M NH_4_OAc (pH 5.0) and the second strip in PBS for each antibody. After the solvent reached the top, the strip was cut in half and each half was measured in a calibrated gamma counter to calculate the percentage of radioactivity that was protein bound, as well as levels of Pb-214 and Bi-214. Bradford protein assay and dose calibrator measurements were also completed on the purified radiolabeled antibodies to quantify the specific activity (μCi activity per μg protein).

### 2.3 Cell culture

The luciferase-positive human ovarian cancer cells SKOV-3 (Catalog #119276) were purchased from Perkin Elmer (Waltham, MD, United States). The luciferase-positive OVCAR-3 cells were purchased from the Japanese Collection of Research Bioresources via Sekisui XenoTech, LLC (Kansas City, KS). SKOV3 cells were grown in McCoy’s media with 10% FBS, 1% Pen/Strep, 1% L-Glutamine and OVCAR3 cells were grown in RPMI, 20% FBS, 1% Pen/Strep, 1% L-Glutamine. All cells were maintained at 37°C and 5% CO_2_ in a humidified incubator.

### 2.4 *In vitro* binding assays

The HER2 binding affinity of Tc-99m-HYNIC-Transtuzumab was determined by saturation binding assays. SKOV3 and OVCAR3 cells were seeded in a 96-well plate and incubated at 37°C and 5% CO_2_ overnight. The next day, cells were washed with PBS and incubated with increasing concentrations of Tc-99m-HYNIC-Trastuzumab, either alone or 100-fold excess unlabeled Trastuzumab, and cells were incubated at 37°C for 1 h. Next, cells were washed with PBS three times, lysed, and ATP levels were measured with the ATPlite kit from Perkin Elmer (Waltham, MA) to determine the cell numbers based on a standard curve. Further, the bound Tc-99m in the cell lysates were counted in an automatic gamma counter. The maximum specific binding (B_max_) and binding affinity (equilibrium dissociation constant, K_d_) were calculated by nonlinear regression curve analysis using GraphPad Prism (GraphPad by Dotmatics, Boston, MA). B_max_ and the number of cells used for this assay were used to calculate the receptors/cells for both SKOV3 and OVCAR3; K_d_ values were also determined. Each experiment was performed in triplicate 3–4 times.

### 2.5 Clonogenic assay

For the clonogenic assay, 1 × 10^3^ luciferase-positive SKOV-3 cells were seeded 2 h prior to the treatment in a 6-well plate. Next, cells were treated with Pb-214/Bi-214-TCMC-Trastuzumab or control Pb-214/Bi-214-TCMC-IgG1 (0.37 MBq/well) in triplicate. After 11 days, D-luciferin (150 μg/mL) was added to each well, and bioluminescence images were collected. Data were recorded using IVIS Spectrum bioluminescence imaging (BLI) with an autoexposure setting. Data were analyzed using ROI and radiance (photons/second).

### 2.6 Animals

Female Balb/c athymic nude mice (8 weeks, strain 194) were purchased from the Charles River Laboratory. The animal studies were approved by the Institutional Animal Care and Use Committee (IACUC) of Michigan State University, United States, and all animal experiments were conducted according to IACUC guidelines.

#### 2.6.1 *In vivo* ovarian tumor therapy studies

Female Balb/c nude mice were implanted intraperitoneally (IP) with 5 × 10^6^ luciferase-positive SKOV-3 cells. After 5 weeks, the mice were randomly assigned to three groups based on the peritoneal bioluminescence signal (n = 5–7/group). Radiolabeled Trastuzumab-anns or IgG1 control antibody was delivered in standard saline (0.9% NaCl). In study 1, mice were injected with one IP dose of 0.74 MBq Pb-214/Bi-214-TCMC-IgG or 0.74 MBq Pb-214/Bi-214-TCMC-Trastuzumab. In study 2, mice were injected with two IP doses of Pb-214/Bi-214-TCMC-IgG or Pb-214/Bi-214-TCMC-Trastuzumab, separated by 7 days. In study 3, 4 × 10^6^ luciferase-positive OVCAR-3 cells were implanted IP in female Balb/c nude mice. After 9 weeks, mice were randomly assigned to three equal groups based on the peritoneal bioluminescence signal (n = 6–7/group) and injected with two IP doses of 0.74 MBq Pb-214/Bi-214-TCMC-IgG or 0.74 MBq Pb-214/Bi-214-TCMC-Trastuzumab on days 1 and 8. Mice were weighed and imaged weekly using an IVIS Spectrum system (Perkin Elmer, Waltham, MD, United States) for 60 days.

Two additional control studies, 4 and 5, were performed using Trastuzumab without radioactivity. Female athymic nude mice (n = 6–7/group) were implanted as described with either luciferase-positive SKOV3 cells (study 4) or OVAR3 cells (study 5) and sorted into two groups for each cell line after either 5 weeks or 9 weeks to match studies 2–3. One group for each cell line was untreated whereas the second group was dosed twice IP with Trastuzumab to match the antibody level of Pb-214/Bi-214-TCMC-Trastuzumab groups from studies 2–3. Mice were weighed and imaged weekly for 60 days.

### 2.7 Statistical analysis

Data were analyzed using GraphPad Prism software (GraphPad Software, Boston, MA). Data are reported as means ± SEM (standard error of the mean). *p*-values <0.05 were considered statistically significant. A *t*-test or one-way analysis of variance (ANOVA) with Dunnett’s test was used to compare the experimental groups to the control group.

## 3 Results

### 3.1 Radon-222 (Rn-222) generator system to produce Pb-214/Bi-214

The manual generator was developed and tested with up to 1.5 GBq Rn-222. ICP-OES analyses of Pb-214/Bi-214 solutions showed low metal impurities (<12 ppm Al; <4 ppm Ag; <2 ppm Cu, Fe, Zn; <1 ppm Pb, Mn, Ni, Cr). Gamma-ray spectroscopy and charged particle spectroscopy analyses revealed no long-lived (t_1/2_ > 5 min), gamma-, β-, or α-particle emitting radioactive impurities outside of an ingrown daughter (<3 Bq Pb-210 per 100 MBq Pb-214), with no detectable activity of Ra-226 (minimum detectable activity <3 Bq per 100 GBq Pb-214).

### 3.2 Radiolabeling, quality controls, and HER2 receptor levels

**FIGURE 2:**
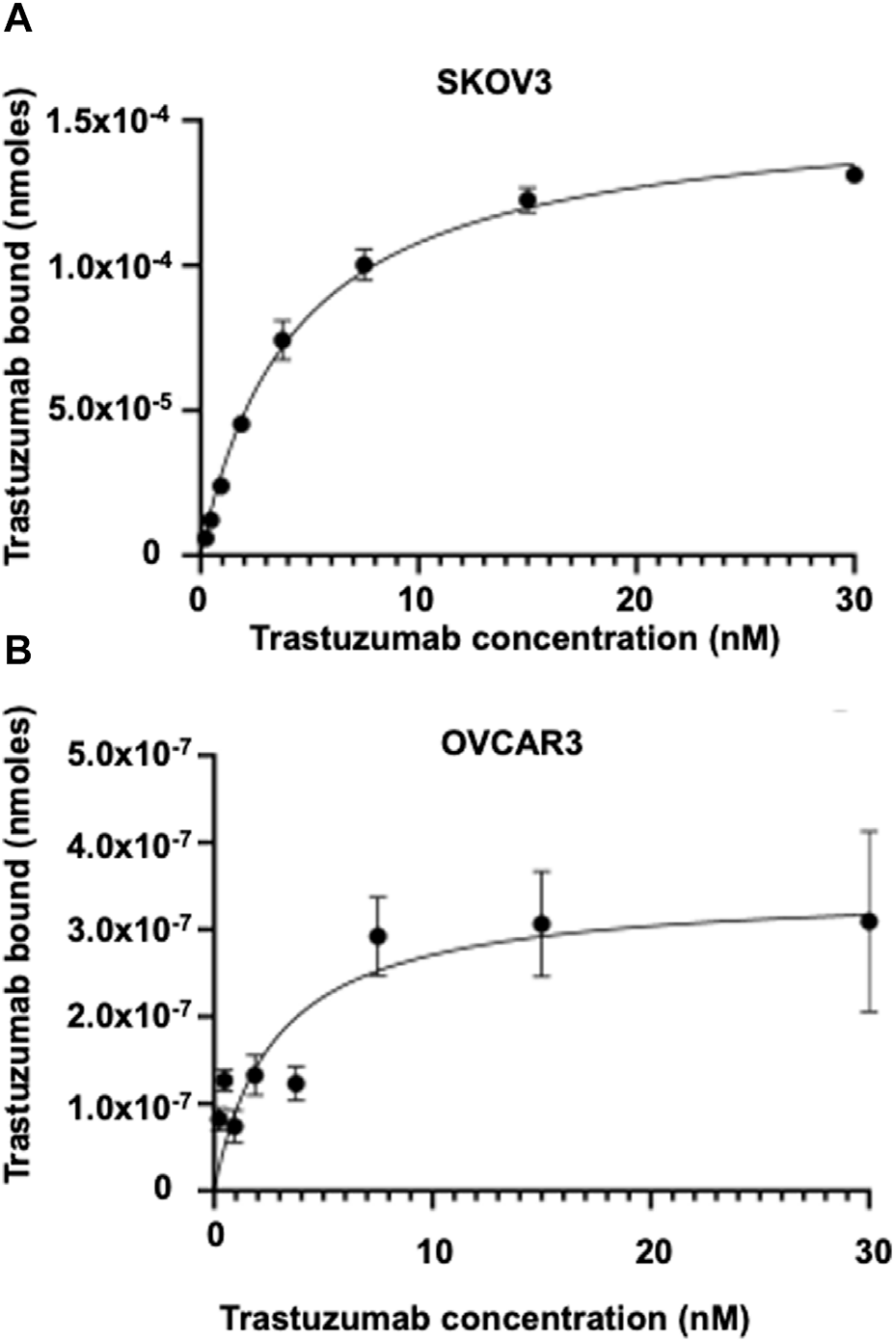
Saturation binding assay showing the binding affinity in HER2 expressing SKOV3 and OVCAR3 cells. Tc-99m radiolabeled Trastuzumab binding affinity and HER2 receptor expression in **(A)** SKOV3 cells and **(B)** OVCAR3 cells.

The HYNIC-conjugated Trastuzumab was successfully radiolabeled with Tc-99m and purity averaged >99%, with a specific activity of 0.37 MBq/μg. The Tc-99m-HYNIC-Trastuzumab binding affinity (K_D_) for SKOV-3 cells was 2 ± 1 nM (Figure 2), which corresponded to 590,000 ± 5,500 HER2 receptors/cell. The Tc-99m-HYNIC-Trastuzumab binding affinity (K_D_) for OVCAR3 cells was 3 ± 1 nM (Figure 2), with 7,900 ± 770 HER2 receptors/cells. These findings established that the SKOV3 cells had ∼75-fold higher HER2 receptors compared to OVCAR3 cells.

TCMC-conjugated Trastuzumab and IgG1 antibodies were successfully radiolabeled with Pb-214/Bi-24 and purified by iTLC in 25 min, with >97% purity. The specific activity ranged from 0.037 to 0.111 MBq/μg. The Bi-214/Pb-214 ratio after purification, as determined from gamma-ray analyses of the iTLC samples, averaged 1.56 ± 0.16 (n = 8). Thus, the 0.74 MBq Pb-214/Bi-214 dose given to the animals contained an average of 0.29 MBq Pb-214 and 0.45 MBq Bi-214.

### 3.3 Effect of Pb-214/Bi-214-TCMC-Trastuzumab treatment on clonogenic survival of SKOV3 cells

**FIGURE 3:**
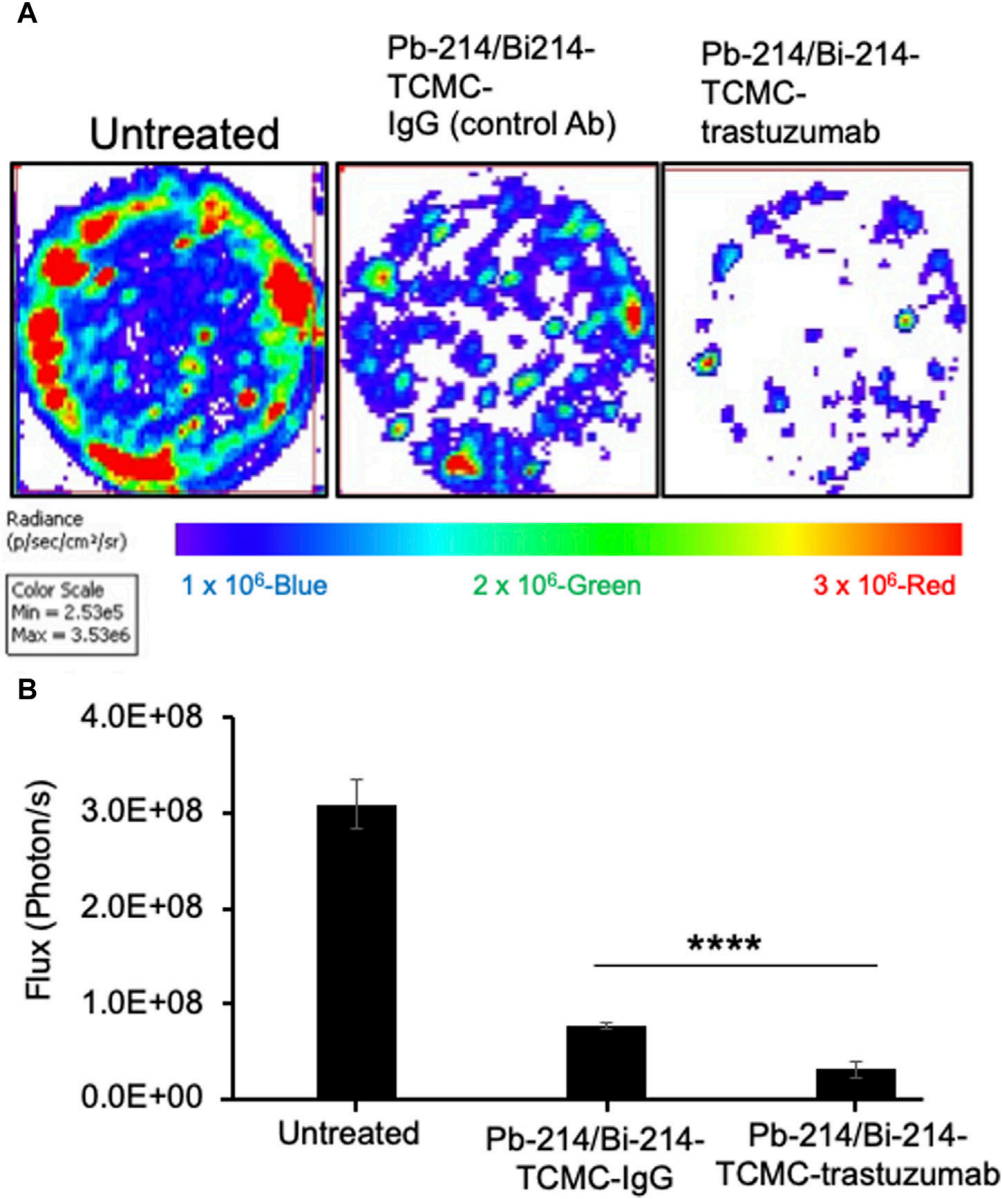
Pb-214/Bi-214-TCMC-Trastuzumab treatment effectively inhibits colony formation in HER2+ SKOV3 ovarian cancer cells **(A,B)**. **(A)** Representative images of three groups (n = 3) and **(B)** summary analyses of living cells showing the Pb-214/Bi-214 response in killing SKOV-3 cells in a clonogenic assay. ****, *p* < 0.0001 compared to untreated control.

In this study, the clonogenic assay was performed to evaluate the efficacy of Pb-214/Bi-214-TCMC-Trastuzumab in suppressing colony formation based on the capacity/survival of HER2+ SKOV3 cells. As shown in Figure 3, the clonogenic assays showed that Pb-214/Bi-214-TCMC-Trastuzumab treated cells had significantly reduced colonies after treatment, with 3.13 × 10^7^ ± 9.36 × 10^6^ total flux (p/s), and Pb-214/Bi-214-TCMC-IgG, with 7.69 × 10^7^ ± 3.39 × 10^6^ total flux (p/s) compared to untreated control groups, with 3.09 × 10^8^ ± 2.55 × 10^7^ total flux (p/s). This finding suggests that TAT using Pb-214/Bi-214-TCMC-Trastuzumab inhibited ovarian cancer cell growth.

### 3.4 TAT using Pb-214/Bi-214-TCMC-Trastuzumab in nude mice with xenografted tumors

**FIGURE 4:**
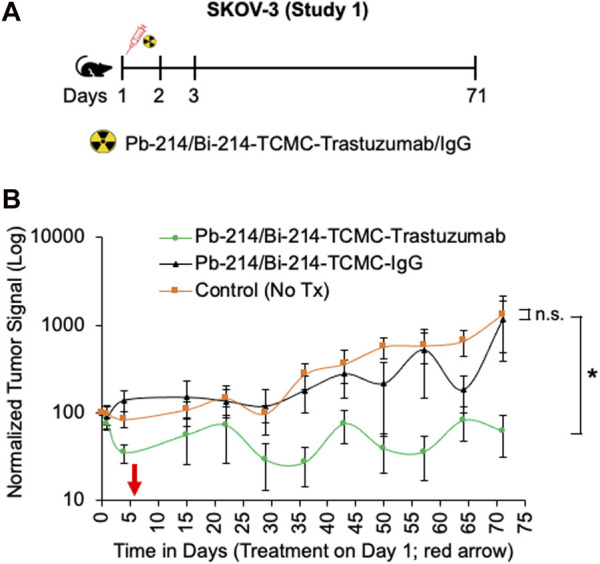
Single intraperitoneal dose of Pb-214/Bi-214-TCMC-Trastuzumab suppresses peritoneal human SKOV3 ovarian tumor growth in a nude mouse model **(A–B)**. **(A)** Design for study #1 and **(B)** summary of bioluminescence imaging results for mice with SKOV3 tumors treated with Pb-214/Bi-214-TCMC-Trastuzumab, Pb-214/Bi-214-TCMC-IgG, and untreated in Balb/c nude mice. *, *p* < 0.05 compared to untreated control; n. s., not significant.

**FIGURE 5:**
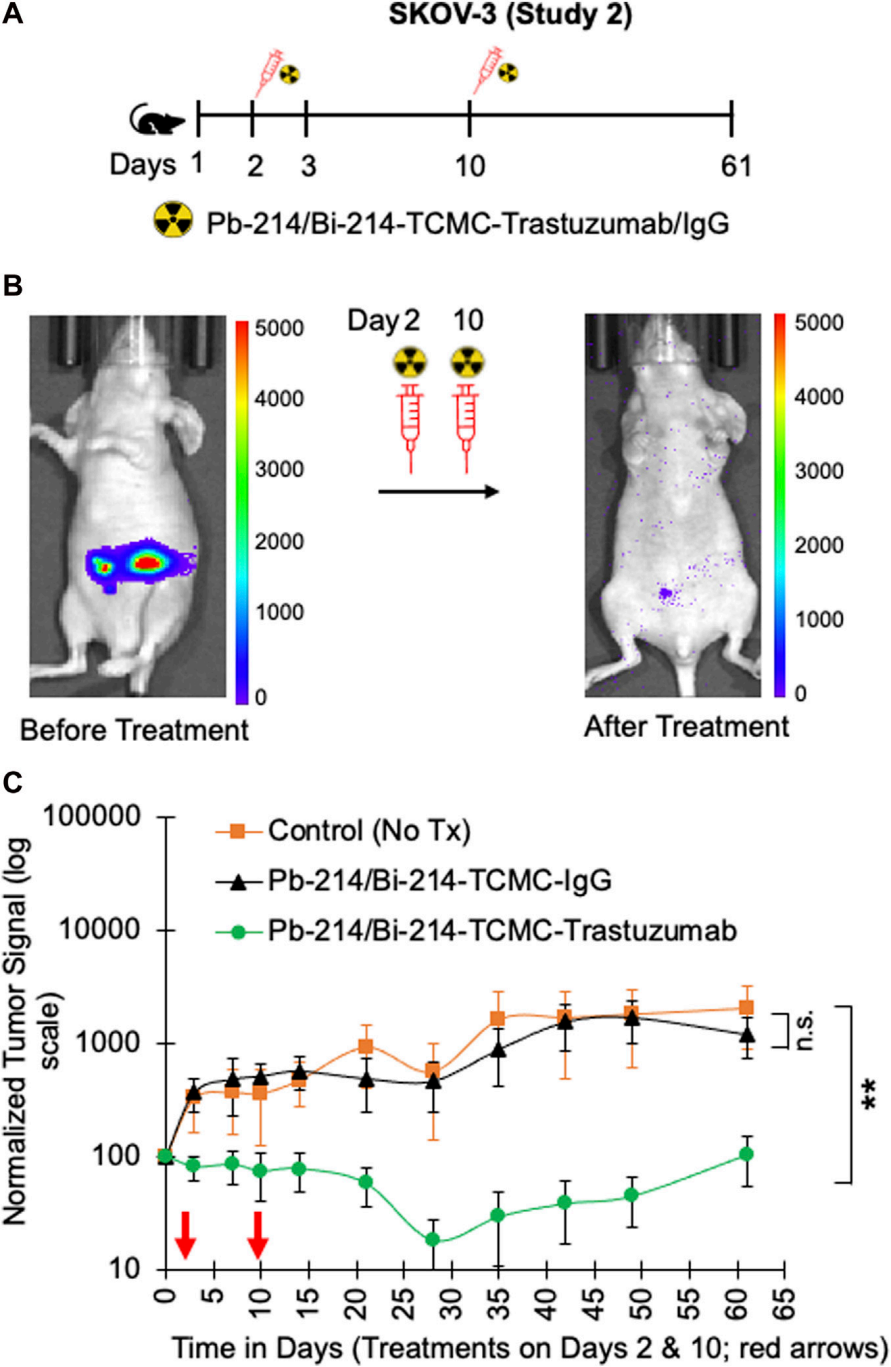
Fractionated intraperitoneal treatments of Pb-214/Bi-214-TCMC-Trastuzumab suppress peritoneal human SKOV3 ovarian tumor growth in a nude mouse model **(A–C)**. **(A)** Design for study #2, **(B)** representative images before and after treatment with Pb-214/Bi214-TCMC-Trastuzumab, and **(C)** summary of bioluminescence imaging results for mice with SKOV3 tumors treated with Pb-214/Bi-214-TCMC-Trastuzumab, Pb-214/Bi-214-TCMC-IgG, and untreated in Balb/c nude mice. **, *p* < 0.01 compared to untreated control; n.s., not significant.

In study 1, a single dose of 0.74 MBq Pb-214/Bi-214-TCMC-Trastuzumab/IgG was given to mice with SKOV3 tumors, and in study 2, two doses of 0.74 MBq Pb-214/Bi-214-TCMC-Trastuzumab/IgG were given (Figure 4A, Figure 5A). In both studies, there was a statistically significant decrease (*p* < 0.05) in tumor growth in the Pb-214/Bi-214-TCMC-Trastuzumab treated group compared to the Pb-214/Bi-214-TCMC-IgG and the untreated groups (Figure 4B, Figures 5B, C). In Study 1, the maximum treatment effect of Pb-214/Bi-214-TCMC-Trastuzumab was observed at day 29 after treatment, while in Study 2 the maximum effect was observed at day 28. At that time in Study 2, the Pb-214/Bi-214-TCMC-Trastuzumab group had an average bioluminescence signal that was 18% of the starting level (100%). In contrast, the Pb-214/Bi-214-TCMC-IgG and untreated groups were significantly higher at 466% and 564%, respectively, of the starting levels. Thus, there was a 5-fold reduction in tumor mass for TAT *versus* approximately a 5-fold increase for the control groups. The untargeted control Pb-214/Bi-214-TCMC-IgG had a slight but not statistically significant effect on tumor growth.

The average body weights of the mice in the three treatment groups were not different over the 60 days (Supplementary Figure S1A) of the treatment period. To address any effect of Trastuzumab alone on tumor growth suppression, study 4 was conducted with athymic nude mice bearing SKOV3 tumors (identical conditions to the TAT study), either untreated or treated with unlabeled Trastuzumab twice (7 µg/dose, equivalent to Trastuzumab in the Pb-214/Bi-214-TCMC-Trastuzumab group) but no tumor suppression was found from this level of Trastuzumab alone (Supplementary Figure S2A).

**FIGURE 6:**
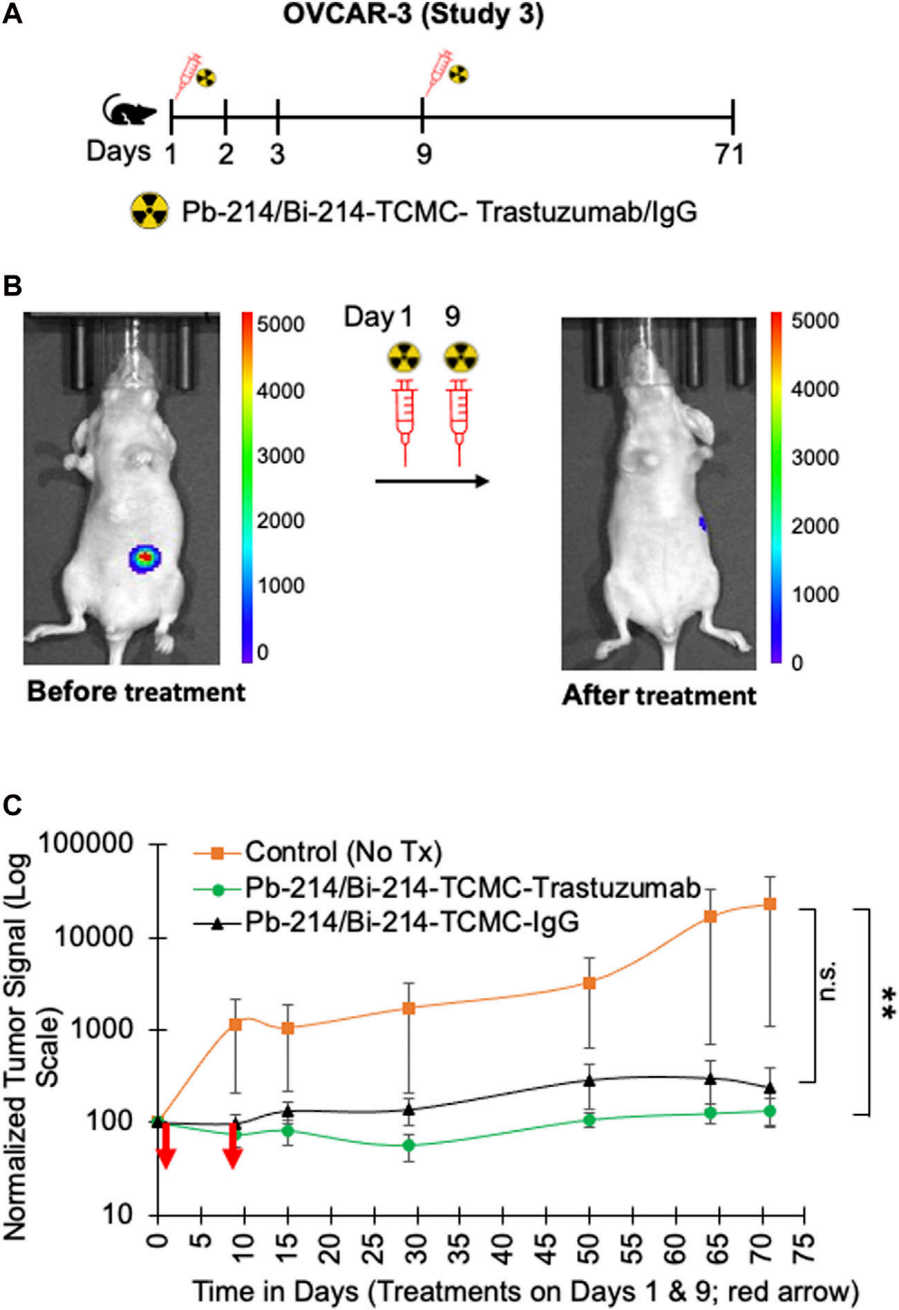
Fractionated intraperitoneal treatments of Pb-214/Bi-214-TCMC-Trastuzumab suppress peritoneal human OVCAR3 ovarian tumor growth in a nude mouse model **(A–C)**. **(A)** Design for study #3, **(B)** representative images before and after treatment with Pb-214/Bi214-TCMC-Trastuzumab, and **(C)** summary of bioluminescence imaging results for mice with OVCAR3 tumors treated with Pb-214/Bi-214-TCMC-Trastuzumab, Pb-214/Bi-214-TCMC-IgG, and untreated in Balb/c nude mice. **, *p* < 0.01 compared to untreated control; n.s., not significant.

In study 3, the nude mice with OVCAR3 tumors were treated with two doses of 0.74 MBq Pb-214/Bi-214-TCMC-Trastuzumab/IgG on days 1 and 9 (Figure 6A). A significant decrease in tumor growth was found for both the Pb-214/Bi-214-TCMC-Trastuzumab and Pb-214-TCMC-IgG treated groups, compared to the untreated control group (Figures 6B, C). By the end of the study, the bioluminescence signal in untreated mice had increased to 254%, compared to the Pb-214/Bi-214-TCMC-Trastuzumab and Pb-214/Bi-214-TCMC-IgG treated groups at 57% and 77%, respectively. OVCAR-3 tumors were in general slower growing than the SKOV3 tumors.

Mice were weighed weekly and the treatment group average weights were not different, as shown in Supplementary Figure S1B. To address any effect of Trastuzumab alone on tumor growth suppression, a separate experiment 5 was conducted with nude mice with OVCAR-3 tumors, either untreated or treated with unlabeled Trastuzumab twice (7 µg/dose, equivalent to Trastuzumab in the Pb-214/Bi-214-TCMC-Trastuzumab group) but no tumor suppression was found from this level of Trastuzumab alone (Supplementary Figures S1B, S2B).

## 4 Discussion

The current study showed that targeted delivery of Pb-214/Bi-214 from a novel generator was able to kill and prevent the growth of both SKOV3 and OVCAR3 tumors in mouse models. Trastuzumab is an FDA-approved antibody to target and block HER2 signaling in breast cancer. Here, Pb-214/Bi-214-TCMC-Trastuzumab was more effective in the treatment of SKOV3 tumors than OVCAR3 tumors, likely because SKOV3 tumors had higher levels of HER2. The data showed that both Pb-214/Bi-214-TCMC-Trastuzumab and Pb-214/Bi-214-TCMC-IgG were effective in treating OVCAR3 tumors that contained 75-fold less HER2 receptors. These findings are explained by the slower growth rate of OVCAR3 tumors compared to SKOV3 tumors, and while the Pb-214/Bi-214-TCMC-IgG did not directly bind the OVCAR3 tumors, it was located near the peritoneal tumors in the peritoneal cavity and thus provided radiation therapy by proximity.

HER2 plays a pivotal role in tumor cell proliferation and metastasis, is found to be implicated in various cancer-poor prognoses, and is associated with aggressive and lethal forms of cancer, including ovarian cancer (Luo et al., 2018; Galogre et al., 2023). Since advanced ovarian cancer has considerably higher expression of HER2, TAT using Trastuzumab could be a promising strategy to reduce the tumor burden for ovarian cancer. However, radiolabeling of an α-particle emitting radionuclide with a very short half-life (Pb-214; t_1/2_ = 26.8 min) for ovarian cancer cells could be challenging. Alpha-particles emitted from the decay of Pb-214/Bi-214 have high linear energy transfer compared to β-particles, which may lead to radiolysis and cause antibody denaturation after radiolabeling before patient dosing. Therefore, rapid radiolabeling requires consideration of various parameters such as optimal pH, temperature, and the potential addition of radiolysis protectants. In this study, we successfully radiolabeled Pb-214/Bi-214 with Trastuzumab using the bifunctional chelator TCMC. We radiolabeled both Trastuzumab and IgG antibodies and purified them in 25 min with a high yield and purity and a specific activity from 0.4 to 1.2 MBq/μg.

In recent years, targeted ɑ-particle therapy has emerged as an option for treating metastatic cancer where treatment modalities such as chemotherapy and radiotherapy using external beams are non-specific for tumors. Additionally, ɑ-particles have an advantage over external beam and β-particles as ɑ-particles produce double-stranded DNA breaks, which are difficult to repair and repair with errors that lead to cancer cell death. However, external beams and β-particles produce single-stranded DNA breaks that can be easily repaired and may lead to radioresistance. Targeted ɑ-particles travel a very short distance compared to β-particles, which makes them a promising candidate for metastatic tumor treatment. Earlier, the ɑ-particle emitting radionuclide Pb-212 had been used for successful treatment of ovarian tumors using intraperitoneal injection by targeting HER2 receptor using Trastuzumab (Meredith et al., 2014; Meredith et al., 2018). However, a toxicological study of Pb-212 in mice showed low-grade toxicity in intravenous and intraperitoneal injections (Milenic et al., 2015). In a dose escalation clinical trial, a dose limiting toxicity was observed for Pb-212-DOTAMTATE in neuroendocrine tumor treatment (Delpassand et al., 2022). Chung et al. showed that Ac-225 ɑ-pre-targeted radioimmunotherapy significantly reduced the SKOV3 tumor in a mouse xenograft model; however, mild toxicity was reported in the kidney (Chung et al., 2023). In the clinical setting, TAT using Ac-225 was associated with hematological and nephrotoxicity in prostate cancer (Parida et al., 2023). In a xenograft mouse model, Bi-213 conjugated with single-domain antibody fragments for the HER2 receptor served as a good vehicle to deliver radiation to peritoneal ovarian tumors and suppressed tumor growth (Dekempeneer et al., 2020). Pb-214/Bi-214 have a very short half-life compared to other ɑ-particle emitting radionuclides; therefore, they may be better for fractionated therapy. Our study showed that Pb-214/Bi-214 fractionated therapy reduced SKOV3 tumors more efficiently compared to a single dose. In this study, Pb-214/Bi-214-TCMC-Trastuzumab treatment significantly reduced the number of colonies in SKOV3 cells, suggesting its cancer cell killing potential in ovarian cancer cells. Further, xenograft ovarian tumor treatment showed the effective killing of tumor cells and suppression of tumor growth by targeted radiotherapy using a 0.74 MBq dose of Pb-214/Bi-214-TCMC-Trastuzumab. These findings suggest that Pb-214/Bi-214 is effective in killing ovarian cancer cells. However, further studies are warranted to evaluate the Pb-214/Bi-214 killing mechanism as well as its treatment efficacy for other metastatic and solid tumors.

The present study is the first report, to the best of our knowledge, of an Rn-222 generator system to harvest Pb-214/Bi-214 for preclinical studies. The Pb-214/Bi-214 generator system should be considered a platform technology that can be applied for both preclinical and clinical studies. Advantages include the isolation of Pb-214/Bi-214 without resins or chemical purification, rather by just moving the Rn-222 gas to another compartment and dissolving the Pb-214/Bi-214 that are decay products of Rn-222. The radiochemical purity of the Pb-214/Bi-214 in our study was better than Pb-212 solutions from typical generator systems and was similar in terms of metal impurities to optimized Pb-212 generator methods (McNeil et al., 2021; McNeil et al., 2023). The Rn-222 generator used to produce Pb-214/Bi-214 may also have a potentially lower cost than other α-particle therapeutics. A lower cost is suggested because the Rn-222 generator can be eluted on an hourly basis instead of daily, similar to what is typical for Pb-212 generator systems. In addition, the parent Rn-222 for the generator is available weekly at Ci levels from current sources of Ra-226. Future studies are aimed at scaling up the Rn-222 generator system to 4 GBq and further optimizing the chemical and radiochemical purity.

The Rn-222 system has many advantages over existing strategies, especially to accomplish fractioned alpha particle therapy and the fact it did not require any resins that may introduce chemical impurities.

Other ɑ-particle emitting radionuclides, including At-211, Ac-225, and Pb-212, are reported to effectively treat different cancers; however, these radionuclides have certain disadvantages, such as the fact that Pb-212 is expensive and not readily available (Meredith et al., 2000; Meredith et al., 2018). Ac-225 has a long half-life and At-211 is not readily available for the development of At-211-labeled radiopharmaceuticals because there are not many cyclotrons available with the 25–30 MeV α-particle beams required for At-211 manufacturing.

In this study, Trastuzumab was radiolabeled with Pb-214/Bi-214 and used for targeted ɑ-particle treatment of ovarian cancer. Our study showed that the α-particle emitting radionuclide Pb-214/Bi-214 can be used to inhibit tumor growth in mouse models using fractionated Pb-214/Bi-214-TCMC-Trastuzumab therapy, suggesting the potential use of Pb-214/Bi-214 for targeted ɑ-particle therapy. In the future, TAT using Pb-214/Bi-214 could be used as a potential radiotherapy modality for metastatic and solid tumors. Moreover, the novel Rn-222 generator system can be eluted hourly to produce Pb-214/Bi-214 with high purity at a potentially lower cost.

## Data Availability

The original contributions presented in the study are included in the article/Supplementary Material, further inquiries can be directed to the corresponding author.

## Supplementary material

SUPPLEMENTARY FIGURE S1 Representative graphs showing mice body weight for **(A)** SKOV3 and **(B)** OVCAR3 tumors treatment with Pb-214/Bi-214-TCMC-Trastuzumab/IgG.

SUPPLEMENTARY FIGURE S1 Representative graphs showing no effect of Trastuzumab treatment on **(A)** SKOV3 and **(B)** OVCAR3 tumors. n.s., not significant.
