# Supplementary figures and images for: Pb-214/Bi-214-TCMC-Trastuzumab inhibited growth of ovarian cancer in preclinical mouse models
Source: Front Chem. 2024 Jan 25;11:1322773. doi: 10.3389/fchem.2023.1322773 (PMC10850308; doi:10.3389/fchem.2023.1322773)

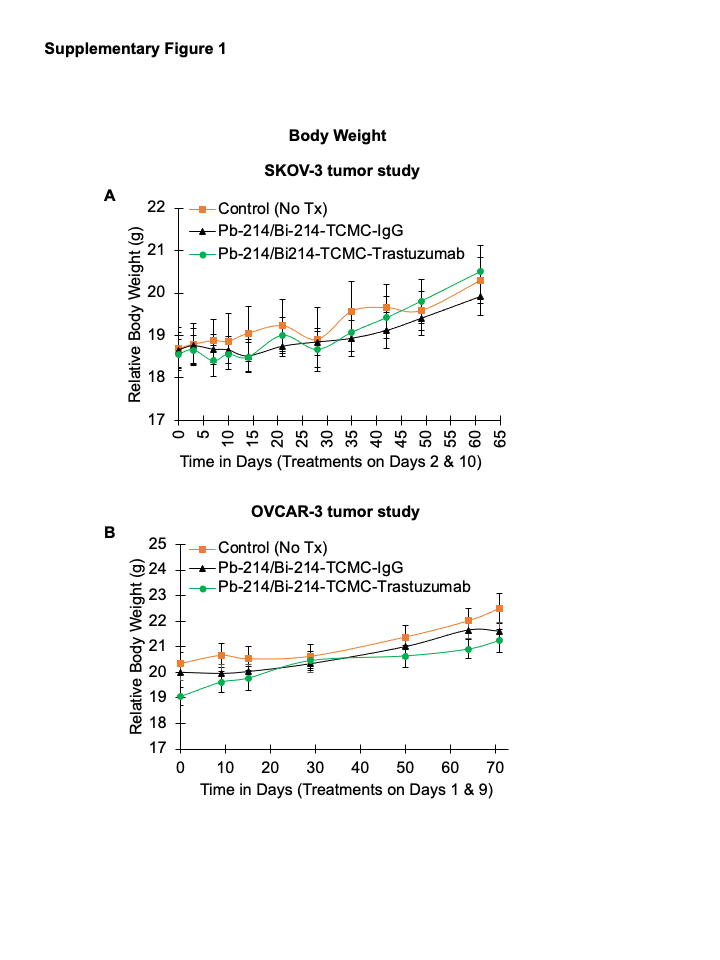

Supplement: Supplementary file 1 [file Image1.TIFF]

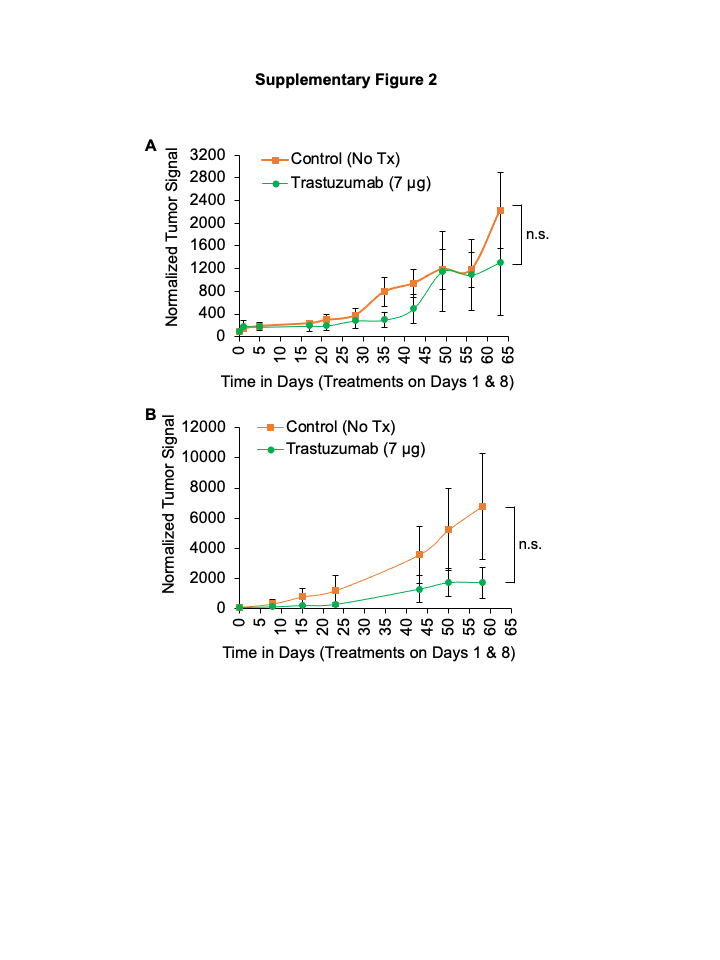

Supplement: Supplementary file 2 [file Image2.TIFF]
